# Evaluation of External Apical Root Resorption in Cases with Extraction and Non-Extraction Fixed Orthodontic Treatment

**DOI:** 10.3390/diagnostics14202338

**Published:** 2024-10-21

**Authors:** Ramazan Berkay Peker, Pamir Meriç

**Affiliations:** 1 Department of Dentomaxillofacial Radiology, Faculty of Dentistry, Trakya University, Edirne 22030, Turkey; 2 Department of Orthodontics, Faculty of Dentistry, Trakya University, Edirne 22030, Turkey; pamirmeric@trakya.edu.tr

**Keywords:** external root resorption, fixed orthodontic treatment, dental radiography, digital panoramic radiography, tooth length

## Abstract

Objective: The objective of this study was to evaluate external apical root resorption (EARR) in cases with extraction and non-extraction fixed orthodontic treatment. Methods: Ninety subjects were included in this study. The patients were divided into two groups: 43 with extraction treatment and 47 with non-extraction orthodontic treatment. EARR was measured using the crown-to-root ratio of the maxillary and mandibular incisors and canines on panoramic radiographs taken at the beginning (T0) and end of the treatment (T1). The Bonferroni corrected Z test was used for multiple comparisons. Results: There were 24 (55.8%) individuals in the extraction group and 12 (25.5%) in the non-extraction group, with a minimum of one tooth with severe resorption. There was no resorption in 0% of individuals in the extraction group and five (10.6%) individuals in the non-extraction group. There was a statistically significant correlation between the groups and the degree of resorption (*p* = 0.008). When the maxillary and mandibular teeth in the extraction group were compared, a significant difference was found in all degrees of resorption except for mild resorption. Conclusions: There was a significant difference in EARR between the extraction and non-extraction treatment groups, with maxillary incisors showing more resorption in the extraction treatment.

## 1. Introduction

External apical root resorption (EARR) is defined as the shortening of the tooth starting from the root apex. The process of EARR begins when, for physiological or pathological reasons, the protective cementoblast layer outside the cement disappears. The higher cellular cementum content on the outer surface of the apical third of the root leads to a higher incidence of external resorption in this area. Many potential risk factors have been identified in the aetiology of EARR. Genetic factors, certain medications, various systemic diseases, and orthodontic movement can all contribute to initiating the formation of EARR [1,2]. Although the effect of orthodontic treatment on EARR is not fully understood, studies comparing the effects of light and heavy orthodontic movements on EARR have shown a positive correlation between heavy orthodontic forces and EARR [3,4]. In addition, it has been reported that EARR seen after orthodontic treatment is mostly in the anterior teeth, while premolars and molars are much less affected [5]. Although cavities formed by EARR can be diagnosed by scanning electron microscopy (SEM), histological sections, and micro-computed tomography methods, tooth extraction is required for these methods [6]. Since panoramic radiography has become the most common and practical radiologic examination technique for diagnostic purposes in dentistry, it has also played an important role in the evaluation of EARR [7].

This retrospective study aimed to compare the effects of extraction and non-extraction orthodontic treatment on root resorption using panoramic radiography.

## 2. Materials and Methods

This retrospective study was approved by the Ethics Committee of Trakya University Faculty of Medicine (Approval Number: TÜTF-GOBEAK 2022/304).

A total of 90 patients who met the inclusion criteria were identified for this study. These patients were divided into two groups: 43 with extraction orthodontic treatment and 47 with non-extraction orthodontic treatment.

The medical records and radiographs of the patients were analysed and examined according to the following inclusion criteria:

- Patients who do not have systemic diseases affecting the jawbones and teeth in their medical records and who do not use medication.
- Patients whose four first premolar teeth had previously been extracted.
- Patients who had orthodontic treatment without extraction.
- Patients with baseline and end-of-treatment panoramic radiographs grade 1 (diagnostically excellent) and grade 2 (diagnostically acceptable), according to the UK National Radiological Protection Board [8].
- Patients with complete root development of all teeth except the second and third molars.
- Patients without periodontal/endodontic lesions on their maxillary and mandibular anterior teeth.
- Patients without endodontic/surgical treatment of their maxillary and mandibular anterior teeth.
- Patients without impacted maxillary and mandibular anterior teeth.
- Patients without dilated roots or morphologic anomalies in maxillary and mandibular anterior teeth.
- Patients without benign or malignant pathology, such as cysts or tumours in their jaws.
- Patients without hypodontia or hyperdontia.
- Patients with no history of orthodontic treatment or trauma.

The sample size was calculated using the G*Power V. 3.1.9.6 program. With 95% confidence (1-α), 80% test power (1-β), p1 = 0.3, and p2 = 0.47, the minimum number of teeth to be included in the study was determined as 374 [9]. A total of 516 teeth were included in this study. According to PostHoc power analysis, the power of the test was obtained as 98.89%.

### 2.1. Orthodontic Intervention

All patients included in this study were treated with 0.22″ McLaughlin Bennett Trevisi (MBT) brackets. Patients who underwent extraction cases with moderate anchorage planning and four first premolar extractions were included in this study. This group’s treatment started with canine distalisation using 17 × 25″ Titanium Molybdenum Alloy (TMA) T loops. After resolving the crowding, extraction spaces were closed with moderate anchorage using 19 × 25″ Stainless Steel (SS) wires. Palatinal root torque was applied to the 19.25″ SS archwire while the maxillary spaces were closed. Class II elastics were used for support during the closure of the extraction spaces, if necessary. In the non-extraction patient group, treatment progressed with standard 0.14″ NiTi, 0.16″ NiTi, 16 × 22″ NiTi, 17.25″ NiTi, 17 × 25″ SS, and 19 × 25″ SS archwires. Interproximal reduction and proclination were performed to gain space.

### 2.2. Radiography and Measurements

Panoramic radiographs were obtained by the same person, with the same standard positioning of each patient, using a Vatech PaX-Flex (Seoul, Republic of Korea) radiography device with parameters of 50–90 kV 4–10 mA 10.1 s. The measurements were performed using computer-aided software such as ImageJ 1.54g (US National Institutes of Health, Bethesda, MD, USA).

**Figure 1:**
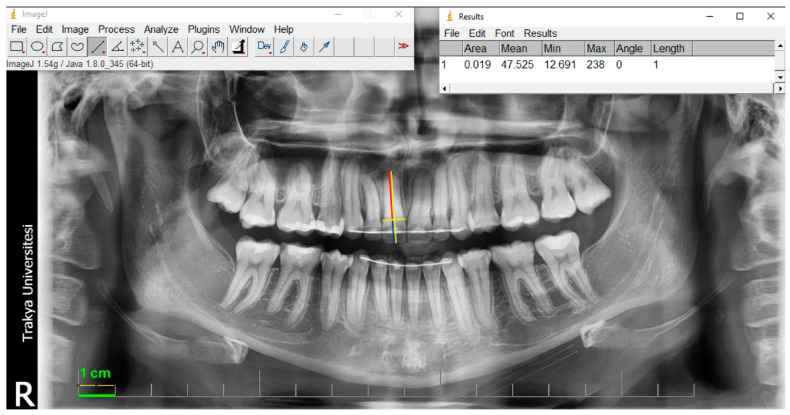
Measurements are taken from a panoramic radiograph. The cementoenamel junction and the long axis of the tooth are represented by yellow lines. The blue line shows the length of the crown, and the red line shows the length of the root.

EARR was measured using the crown-to-root ratio of the maxillary and mandibular incisors and canines on panoramic radiographs taken at the beginning (T0) and end of the treatment (T1). The method used by Gay et al. in their study was used to calculate the EARR ratio [10]. While exporting the panoramic images, the automatic calibration tab in the automation program was clicked. In this way, a reference ruler was created on the panoramic image. Then, using the reference ruler on the panoramic image, calibration was performed in ImageJ software based on the previously known distance (1 cm). Although the panoramic radiographs showed different magnification patterns, no additional calibration was performed because the crown-to-root ratio remained constant (Figure 1). 

**Figure 2:**
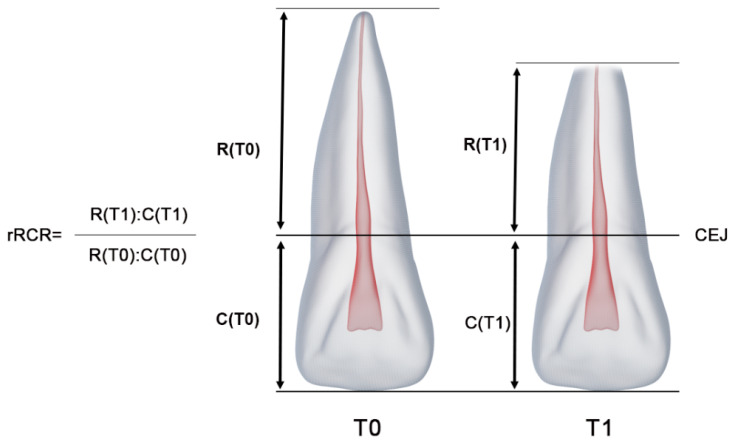
Determination of pre-treatment and post-treatment root and crown lengths in panoramic radiographs. The formula for calculation of changes in root length: pre-treatment (T0), post-treatment (T1), pre-treatment crown length (C[T0]), post-treatment crown length (C[T1]), pre-treatment root length (R[T0]), post-treatment root length (R[T1]), the cementoenamel junction (CEJ), and relative changes of root-to-crown ratio (rRCR).

While measuring EARR, a line was created extending from the root apex of each tooth to the incisal edge and parallel to the long axis. This line was bisected by another line connecting the mesial and distal cementoenamel junction (CEJ). The line between the CEJ and the root apex was defined as the root length (R), and the line between the CEJ and the incisal edge was defined as the crown length (C). The root–crown ratio (RCR) was calculated by dividing the R-value of each tooth at the beginning and end of treatment by the C-value of each tooth at the beginning and end of treatment. The relative root-to-crown ratio (rRCR), which describes the percentage change in root length during treatment, was calculated by dividing the RCR value at the end of treatment by the RCR value at the beginning of treatment. The rRCR scale used by Gay et al. was used in our study [10]. According to this scale, rRCR ≥ 100% was scored as no EARR, rRCR = 90–99% as mild EARR, rRCR = 80–90% as moderate EARR and rRCR < 80% as severe EARR (Figure 2). A calibration session between an experienced dentomaxillofacial radiologist (RBP) and an experienced orthodontist (PM) was performed before the measurements were taken. To prevent bias, group assignments to the dentomaxillofacial radiologist and orthodontist were both random and blinded. 

### 2.3. Statistical Method

Data were analysed using IBM SPSS V23. The conformity of the data to a normal distribution was examined using the Shapiro–Wilk test. The independent samples *t*-test was used to compare the data that showed a normal distribution according to paired groups. The Mann–Whitney U test was used to compare data that did not show a normal distribution according to the binary groups. Yates correction, the Fisher–Freeman–Halton test, and the Pearson chi-square test were used to compare categorical data. The Bonferroni corrected Z test was used for multiple comparisons. Analysis results were presented as frequency (percentage) for categorical variables, mean ± standard deviation, and median (minimum–maximum) for quantitative variables. The significance level was taken as *p* < 0.050.

## 3. Results

There was no statistically significant difference between the median age values at the beginning of the treatment (*p* = 0.336). There was a statistically significant difference between the median values of treatment duration (*p* < 0.001). The mean duration of treatment was 3.1 years in the extraction group and 2.4 years in the non-extraction group. There was no statistically significant correlation between group and gender (*p* = 1.000; Table 1). In the extraction group, 0% of individuals had no resorption, 14% had mild resorption, 30.2% had moderate resorption, and 55.8% had severe resorption (Table 2). In the non-extraction group, 10.6% of individuals had no resorption, 19.1% had mild resorption, 44.7% had moderate resorption, and 25.5% had severe resorption. There was a statistically significant correlation between the groups and the degree of resorption (*p* = 0.008). The groups differed between those with no resorption and those with severe resorption.

Descriptive statistics of the degree of tooth resorption according to groups are presented in Table 3.

When the total number of teeth between the extraction and non-extraction groups were analysed according to the degree of resorption, 46.3% of teeth in the extraction group had no resorption, 25.6% had mild resorption, 19.2% had moderate resorption, and 8.9% had severe resorption (Table 4). In the non-extraction group, 65.4% of teeth had no resorption, 20% had mild resorption, 12.2% had moderate resorption, and 2.3% had severe resorption. There was a statistically significant correlation between the groups and the degree of resorption (*p* < 0.001). Groups differed in all degrees. In the extraction group, a comparison of the degree of resorption according to jaw showed that 36.8% of the maxillary teeth had no resorption, 26.7% had mild resorption, 23.3% had moderate resorption, and 13.2% had severe resorption (Table 5). In the mandibular teeth, 55.8% had no resorption, 24.4% had mild resorption, 15.1% had moderate resorption, and 4.7% had severe resorption. There was a statistically significant relationship between jaw and the degree of resorption (*p* < 0.001). This difference was due to the groups with no resorption, moderate resorption, and severe resorption. In the non-extraction group, there was no statistically significant correlation between the jaw and the degree of resorption (*p* = 0.055).

A statistically significant very high agreement was obtained between inter-observer measurements (ICC = 0.948; *p* < 0.001; Table 6).

## 4. Discussion

Crown and root lengths can be measured using various radiographic techniques. Studies have shown that cone beam computed tomography (CBCT) and periapical radiography are effective in the examination of EARR caused by orthodontic forces [11,12,13,14,15]. However, the high dose of ionizing radiation when using CBCT imaging for diagnosis and treatment limits the use of this technique. Although the relationship between radiation dose and biological damage is not fully understood, studies are showing that high radiation doses are associated with cancer development during adolescence [16,17]. In addition, CBCT imaging is only used for specific indications. It is unethical to use CBCT imaging solely to obtain study data. Therefore, in our study, panoramic radiographs were preferred for the evaluation of the available data to prevent the repeated exposure of individuals to radiation since panoramic radiographs are routinely used at the beginning and end of orthodontic treatments. Linear measurements on panoramic radiographs have been reported to be safe when there is a lateral inclination of 10° or less in the occlusal plane [18]. Panoramic radiographs show magnification in different ways. Since the crown-to-root ratio, not the crown-to-root length, was analysed in this study, there was no magnification effect, and no special calibration was required.

External apical root resorption (EARR) is a common iatrogenic condition associated with orthodontic treatment. Root resorption of more than 3 mm is seen in more than one-third of orthodontically treated patients, and severe root resorption of more than 5 mm is seen in 2–5% of the population [11,12,13]. The duration of orthodontic treatment and applied forces are considered the primary causes of EARR [14,15,16,17]. However, age, gender, type of malocclusion, root morphology, application of retraction mechanics in extraction cases, and genetic polymorphism have been associated with the risk of EARR [14,15,16,18,19]. EARR mostly affects maxillary central and lateral incisors, followed by mandibular incisors [9]. This may be due to their single cone-shaped roots. Therefore, we evaluated the EARR of the maxillary and mandibular incisors and canines in our study.

The principal finding of our study was that there was a statistically significant difference between extraction and non-extraction cases at all resorption severities. The number of root resorptions was higher in the extraction group. Studies comparing extraction and non-extraction treatments have found results similar to ours [20,21]. However, in the extraction group, when the maxillary and mandibular teeth were compared, a significant difference was found in all degrees of resorption except for mild resorption. The amount of EARR was found to be higher in the maxillary teeth than in the mandibular teeth. In the non-extraction group, the difference between the maxillary and mandibular teeth was not statistically significant. It has been reported that upper incisors are more prone to root resorption because of their single and cylindrical root structures and because they move more than posterior teeth during orthodontic treatment [22]. However, there are differences in the mechanics applied in extraction and non-extraction treatments; there may be an increase in root resorption in incisors due to the movement of the incisor roots towards the palatal cortical bone [23,24]. This may be one of the reasons why more severe resorption is seen in extraction cases than in non-extraction cases. Another reason the upper incisors are affected may be the application of torque and intrusive movements during the retraction of the upper incisors in extraction cases. In previous studies, EARR was found more frequently when retraction and intrusive movements were combined in extraction cases than when only retraction was performed [25,26,27]. This is because the intrusive forces [28] and palatinal root torque [25,27,29] applied at the stage of closing the extraction space [30,31] cause excessive loads on the incisor apices. However, there may be a difference in the severity of resorption between the upper incisors and the lower incisors since a large amount of incisor retraction is usually not performed after extractions to relieve crowding in the mandible [32]. This supports our findings.

There are studies in the literature on the EARR of different orthodontic appliances. In non-extraction treatments in which the effects of clear aligners and pre-adjusted brackets on root resorption were examined, it was found that clear aligners caused less root resorption [33]. This is due to the following reasons: aligners might not be used for the recommended number of hours, clear aligners are less preferred in extraction cases requiring complex tooth movement, and the cementum can begin the self-repair process due to the lack of continuous force application [34]. However, compared to fixed orthodontic treatment, round-tripping movements can be seen less in clear aligner treatments since the direction of tooth movement is planned. Round-tripping movements may increase root resorption and prolong treatment duration [20,35]. In a study using self-ligating and conventional metal brackets, although the incidence of slanted resorption was higher in the conventional bracket group, it was not possible to conclude that the self-ligating bracket system was superior to conventional brackets [36]. In a study comparing lingual and labial fixed orthodontic appliances used in the non-extraction treatment group, similar levels of resorption were found in both groups [9]. In our study, all patients received conventional labial metal braces. It has been shown in the literature that the open apex and closed apex react differently to root resorption [37]. Teeth with incomplete root apices showed higher biological tolerance to resorption. Even with high forces, a wide-open apex exposes the tooth to fewer circulatory problems. Immature, non-calcified tissues resist root resorption better than calcified forms [37]. In our study, the root apex was closed in all teeth in which force was applied.

### Limitations of the Study

One limitation of our study is the use of 2D panoramic radiography instead of 3D imaging techniques to examine EARR. However, 3D imaging should be used for specific indications for the benefit of the patient. Another limitation is that only patients with labial fixed orthodontic treatments were included in this study.

## 5. Conclusions

Based on our findings, a statistically significant difference was found between the extraction and non-extraction groups in terms of EARR. The number of severe, moderate, and mild resorptions in the teeth evaluated in the extraction treatment group was higher than in the non-extraction group. In the extraction treatment group, the number of teeth affected by severe and moderate resorption was higher in the maxillary teeth. There was no difference in the degree of resorption between the maxillary and mandibular teeth in the non-extraction treatment group.

## Figures and Tables

**Table 1:** Comparison of demographic data according to the groups.

	Group		Total	Test Statistics	*p*
	Extraction	Non-Extraction			
Age (years)	16.1 ± 3.1	15.5 ± 2.3	15.8 ± 2.7	891.5	0.336 *
	15.6 (11.4–28.9)	15.3 (11.7–22.8)	15.6 (11.4–28.9)		
Treatment duration (years)	3.1 ± 0.8	2.4 ± 0.6	2.7 ± 0.8	77.409	<0.001 **
	3.2 (1.4–4.8)	2.3 (1.2–3.8)	2.7 (1.2–4.8)		
Gender	*n* (%)	*n* (%)	*n* (%)		
Male	9 (20.9)	10 (21.3)	19 (21.1)	0	1.000 ***
Female	34 (79.1)	37 (78.7)	71 (78.9)		

* Mann Whitney U test; ** Independent Samples *t*-test; *** Yates Correction; Mean ± Standard Deviation; Median (Minimum–Maximum).

**Table 2:** Comparison of the number of individuals in the extraction and non-extraction treatment groups according to the degree of resorption.

	Group		Total *n* (%)	Test Statistics	*p* *
	Extraction *n* (%)	Non-Extraction *n* (%)			
Resorption degree					
No resorption	0 (0) ^a^	5 (10.6) ^b^	5 (5.6)	11.128	0.008
Mild resorption	6 (14)	9 (19.1)	15 (16.7)		
Moderate resorption	13 (30.2)	21 (44.7)	34 (37.8)		
Severe resorption	24 (55.8) ^a^	12 (25.5) ^b^	36 (40)		
Total	43 (100)	47 (100)	90 (100)		

* Fisher–Freeman–Halton test; ^a,b^: No difference between groups with the same letter.

**Table 3:** Descriptive statistics of the degree of tooth resorption according to groups.

		No Resorption		Mild Resorption		Moderate Resorption		Severe Resorption		Total	
Group	Tooth Number	*n*	%	*n*	%	*n*	%	*n*	%	*n*	%
Extraction	11	16	37.2	13	30.2	8	18.6	6	14	43	100
	12	17	39.5	7	16.3	10	23.3	9	20.9	43	100
	13	9	20.9	14	32.6	13	30.2	7	16.3	43	100
	21	24	55.8	12	27.9	5	11.6	2	4.7	43	100
	22	17	39.5	13	30.2	10	23.3	3	7	43	100
	23	12	27.9	10	23.3	14	32.6	7	16.3	43	100
	31	28	65.1	11	25.6	4	9.3	-	-	43	100
	32	30	69.8	8	18.6	4	9.3	1	2.3	43	100
	33	25	58.1	12	27.9	3	7	3	7	43	100
	41	23	53.5	12	27.9	6	14	2	4.7	43	100
	42	20	46.5	11	25.6	9	20.9	3	7	43	100
	43	18	41.9	9	20.9	13	30.2	3	7	43	100
	Total	239	39.3	132	53.9	99	58.9	46	78		
Non-extraction	11	24	51.1	11	23.4	10	21.3	2	4.3	47	100
	12	28	59.6	9	19.1	8	17	2	4.3	47	100
	13	32	68.1	8	17	7	14.9	-	-	47	100
	21	26	55.3	13	27.7	7	14.9	1	2.1	47	100
	22	31	66	9	19.1	5	10.6	2	4.3	47	100
	23	30	63.8	10	21.3	5	10.6	2	4.3	47	100
	31	30	63.8	11	23.4	5	10.6	1	2.1	47	100
	32	30	63.8	12	25.5	5	10.6	-	-	47	100
	33	40	85.1	5	10.6	1	2.1	1	2.1	47	100
	41	30	63.8	12	25.5	5	10.6	-	-	47	100
	42	29	61.7	7	14.9	11	23.4	-	-	47	100
	43	39	83	6	12.8	-	-	2	4.3	47	100
	Total	369	60.7	113	46.1	69	41.1	13	22		

**Table 4:** Comparison of the total number of teeth between the extraction and non-extraction groups according to the degree of resorption.

	Group		Total *n* (%)	Test Statistics	*p* *
	Extraction *n* (%)	Non-Extraction *n* (%)			
Resorption degree					
No resorption	239 (46.3) ^a^	369 (65.4) ^b^	608 (56.3)	51.052	<0.001
Mild resorption	132 (25.6) ^a^	113 (20) ^b^	245 (22.7)		
Moderate resorption	99 (19.2) ^a^	69 (12.2) ^b^	168 (15.6)		
Severe resorption	46 (8.9) ^a^	13 (2.3) ^b^	59 (5.5)		
Total	516 (100)	564 (100)	1080 (100)		

* Pearson chi-square test; ^a,b^: No difference exists between groups with the same letter.

**Table 5:** Comparison of the degree of teeth resorption between maxilla and mandible in extraction and non-extraction treatment groups.

Group											
Extraction						Non-Extraction					
			Total	Test Statistics	*p* *				Total	Test Statistics	*p* *
	Mx	Mnd					Mx	Mnd			
Resorption degree	*n* (%)	*n* (%)	*n* (%)			Resorption degree	*n* (%)	*n* (%)	*n* (%)		
No resorption	95 (36.8) ^a^	144 (55.8) ^b^	239 (46.3)	25.295	<0.001	No resorption	171 (60.6)	198 (70.2)	369 (65.4)	7.593	0.055
Mild resorption	69 (26.7)	63 (24.4)	132 (25.6)			Mild resorption	60 (21.3)	53 (18.8)	113 (20)		
Moderate resorption	60 (23.3) ^a^	39 (15.1) ^b^	99 (19.2)			Moderate resorption	42 (14.9)	27 (9.6)	69 (12.2)		
Severe resorption	34 (13.2) ^a^	12 (4.7) ^b^	46 (8.9)			Severe resorption	9 (3.2)	4 (1.4)	13 (2.3)		
Total	258 (100)	258 (100)	516 (100)			Total	282 (100)	282 (100)	564 (100)		

* Pearson chi-square test; ^a,b^: No difference exists between groups with the same letter. Mx: Maxilla, Mnd: Mandible

**Table 6:** Examination of inter-observer agreement in measurement values.

	ICC (%95 CI)	*p*
Measurements	0.948 (0.927–0.963)	<0.001

CI: Confidence Interval.

## Data Availability

The original contributions presented in the study are included in the article, further inquiries can be directed to the corresponding author.
